# The impact of creativity on functional outcome in schizophrenia: a mediational model

**DOI:** 10.1038/s41537-021-00144-5

**Published:** 2021-02-26

**Authors:** Agurne Sampedro, Javier Peña, Pedro Sánchez, Naroa Ibarretxe-Bilbao, Nagore Iriarte-Yoller, Cristóbal Pavón, Isabel Hervella, Mikel Tous-Espelosin, Natalia Ojeda

**Affiliations:** 1 Department of Methods and Experimental Psychology, Faculty of Psychology and Education, University of Deusto, Bilbao, Spain; 2 Refractory Psychosis Unit, Hospital Psiquiátrico de Alava, Vitoria, Spain; 3 Department of Neuroscience, Psychiatry Section, School of Medicine and Odontology, University of the Basque Country (UPV/EHU), Vizcaya, Spain; 4 Department of Physical Education and Sport, Faculty of Education and Sport, University of the Basque Country (UPV/EHU), Vitoria-Gasteiz, Spain

**Keywords:** Schizophrenia, Psychosis

## Abstract

Functional impairment remains one of the most challenging issues for treatment in schizophrenia. However, previous studies have mainly focused on the negative impact of symptoms excluding variables that could positively impact functional outcome, such as creativity, which is considered an adaptive capacity for real-life problem-solving. This study analyzed the predictive role of creativity on functional outcome in 96 patients with schizophrenia through a mediational model, including sociodemographic, clinical, neurocognitive, and social cognitive variables. Path analysis revealed that creativity significantly mediated the relationship between neurocognition and functional outcome, and that creativity mediated between negative symptoms and functional outcome. Additionally, neurocognition was directly associated with functional outcome and social functioning was associated with creativity. The involvement of creativity in functional outcome could have relevant implications for the development of new interventions. These findings open up a new field of research on additional personal resources as possible factors of functional outcome in schizophrenia and other diseases.

## Introduction

In spite of advances in pharmacological and psychological treatments, schizophrenia is still considered one of the most disabling disorders in the world^1^. Patients with schizophrenia experience difficulties in a variety of daily life functioning domains, mainly in independent living, occupational functioning, and social functioning^2^. This functional impairment not only affects the patients’ quality of life, but also has indirect effects on their relatives and caregivers^3^. Furthermore, this impairment leads to a very high financial cost^4^. For all these reasons, it is necessary to correctly identify the factors that hinder functional outcome in this disease in order to establish better potential treatment targets. Indeed, in the last two decades, there has been a burst of interest in studying factors underlying functional impairment of people with schizophrenia^4^. The main factors that have been proposed are cognitive impairment and severity of clinical symptoms^5–9^.

Regarding clinical symptoms, both negative and positive symptoms have been related to functional outcome^1,10,11^, although the former seems to have a greater impact^6,12–14^. Moreover, primary negative symptoms seem to contribute to functional outcome after controlling for secondary negative symptoms such as those related to medication^10^. Specifically, motivational deficits have shown to be the most robust predictors of functional outcome^15^. Another clinical factor that has also been related to functional outcome is disorganization, which is closely related to neurocognition^1^.

With respect to cognition, cognitive deficits in neurocognition and social cognition seem to be a core feature of this disease^16^. Numerous studies have shown that cognitive functioning is a main predictor of functional outcome in schizophrenia^1,6,8,9,17–20^. Interestingly, a meta-analysis carried out by Fett et al.^21^ found that social cognition had a stronger involvement in functional outcome than neurocognition. Furthermore, evidence from several studies suggests that social cognition could act as a mediator between neurocognition and functional outcome^1,22–24^. Additionally, clinical symptoms, mainly negative symptoms, have also been suggested to mediate between cognition and functional outcome^6,12,13^.

Besides these clinical and cognitive factors, it has been suggested that other kinds of personal resources could also be important determinants of real-life functioning^1,11^. In particular, creative capacity could be crucial for functional outcome in schizophrenia^25^. Creativity consists of the capacity to produce something original or novel and appropriate or useful for a task^26^. Evidence from studies carried out in the general population indicates that creativity is a key component for real-life problem-solving^27^ and it is considered an essential resource for adaptation and coping with daily life adversities in healthy people^28,29^. This capacity increases the ability to perform everyday activities through the application of creative problem-solving skills to daily life problems^30^. Moreover, creativity applied to our daily life has been shown to improve individuals’ physical and psychological health as well as self-competency, life satisfaction, social life, civil participation, and academic and job performance^31–33^. Regarding creativity in schizophrenia, a recent meta-analysis indicates that people with this disease show a worse creative performance than the general population^34^. Nemoto et al.^25^ found higher creativity to be related to better functional outcome in schizophrenia. Specifically, these authors found that verbal creativity (measured through an idea fluency test)^35^ was related to daily living and community functioning and figural creativity (measured through a design fluency test)^35^ was associated with interpersonal relations and community functioning^25^. In this study, in addition to creativity, negative symptoms and general psychopathology predicted functional outcome, but not neurocognition^25^. In another study, nonverbal creativity was related to different coping strategies within schizophrenia spectrum and bipolar disorder^36^. Additionally, in a pilot study, a creativity intervention was shown to improve negative symptoms, creativity, general psychopathology, global functioning, and interpersonal relations^37^. Likewise, research carried out among the elderly and people with bipolar disorder have suggested that creativity may act as a compensatory advantage for better daily functioning and well-being^31,32^. Thus, evidence suggests that those patients with schizophrenia who have better creative skills may possibly benefit from this resource for better and more adaptive daily life functioning.

Although creativity has been defined as a complex multidimensional construct, it is not a completely independent variable in schizophrenia. Previous literature suggests that creativity is closely related to neurocognition and social cognition in schizophrenia^38–42^. Specifically, both social cognition and neurocognition have been shown to play a mediatory role in the relationship between creativity and schizophrenia^40^. Additional evidence comes from studies that have found the metacognitive ability (which is related to creative thinking)^43,44^ to be associated with functional outcome in schizophrenia^45,46^ as well as to mediate the relationship between neurocognition and functional outcome in this disease^47^. In addition to cognition, clinical symptoms, specifically negative symptoms, have also been shown to predict creativity^39,41^.

Altogether, the literature suggests that functional outcome in schizophrenia may be explained not only by factors such as clinical symptoms and cognitive impairment, but also by other factors like creative ability. However, as far as the authors are aware, to date no study has explored the predictive value of creativity on functional outcome in this disease through a mediational model including clinical symptoms, neurocognition, and social cognition. Therefore, the aim of this study was to analyze the contribution of creativity in functional outcome of patients with schizophrenia through a mediational model analyzing the interplay of clinical symptoms, neurocognition, social cognition, and creativity when explaining their contribution to functional outcome. Based on previous evidence, it was hypothesized that creative capacity would be associated with functional outcome, mediating the association between neurocognition, social cognition, negative symptoms, and functional outcome in schizophrenia.

## Results

The average performance scores on clinical, neurocognitive, social cognitive, creativity, and functional outcome tests are shown in Table 1. To check the association between sociodemographic data and the rest of the variables of the study, correlation analyses were performed (Table 2). Additionally, correlations between neurocognitive individual domains, social cognitive domains, creativity, and functional outcome are shown in Supplementary Table 1. Several statistically significant associations were found. Regarding sociodemographic variables, sex (being female) correlated significantly and negatively with negative symptoms (*r*_pb_ = −0.261, *p* = 0.010) and excitement (*r*_pb_ = −0.237, *p* = 0.020). Age correlated significantly and negatively with neurocognition (*r* = −0.386, *p* < 0.001) and social cognition (*r* = −0.334, *p* = 0.001), and positively with duration of illness (*r* = 0.856, *p* < 0.001). Finally, years of education correlated significantly and negatively with disorganization (*r*_s_ = −0.260, *p* = 0.010) and excitement (*r*_s_ = −0.221, *p* = 0.031), and positively with neurocognition (*r*_s_ = 0.317, *p* = 0.002) and social cognition (*r*_s_ = 0.254, *p* = 0.013).

**Table 1:** Descriptive data of clinical, neurocognitive, social cognitive, creativity, and functional outcome measures.

	*M (SD)*
Clinical characteristics	
PANSS Positive	9.98 (4.22)
PANSS Disorganization	7.78 (2.67)
PANSS Excitement	7.92 (3.71)
PANSS Depression	6.37 (2.41)
BNSS	31.67 (14.14)
Neurocognition	
M-WCST categories	3.24 (1.78)
M-WCST perseverative errors	7.41 (8.13)
Stroop Word	90.47 (18.94)
Stroop Color	57.13 (14.25)
Stroop Word-Color	33.47 (10.90)
Backward Digit Span	4.96 (1.51)
HVLT three learning trials	17.46 (4.81)
HVLT delayed recall	5.02 (2.45)
Symbol-Coding	45.98 (15.28)
Social cognition	
Happé test	4.43 (2.45)
SAT-MC-II	9.34 (4.36)
BLERT	14.83 (3.87)
Creativity	
Figural creativity	48.75 (19.56)
Figural strengths	2.47 (2.35)
Verbal creativity	17.73 (9.18)
Functional outcome	
SFS (short version)	23.45 (5.01)

*M* = Mean, *SD* = Standard deviation, *PANSS* = Positive and Negative Syndrome Scale, *BNSS* = Brief Negative Symptom Scale, *M-WCST* = Modified Wisconsin Card Sorting Test, *HVLT* = The Hopkins Verbal Learning Test, *SAT-MC-II* = Social Attribution Task-Multiple Choice II; *BLERT* = Bell Lysaker Emotion Recognition Test, *SFS* = Social Functioning Scale.

**Table 2:** Correlations between sociodemographic variables with clinical characteristics, neurocognition, social cognition, creativity, and functional outcome.

	Sex (female)	Age	Education (years)	Handedness
Duration of illness (years)	−0.011	0.856**	−0.150	0.059
Negative symptoms	−0.261*	0.012	−0.154	0.153
Positive symptoms	−0.121	−0.101	−0.146	0.053
Disorganization	−0.159	0.039	−0.260*	0.183
Excitement	−0.237*	−0.115	−0.221*	0.035
Depression	−0.047	−0.074	0.038	0.135
Neurocognition	0.015	−0.386**	0.317*	−0.037
Social cognition	−0.058	−0.334**	0.254*	−0.188
Figural creativity	0.122	−0.064	0.105	−0.133
Figural strengths	−0.017	−0.141	0.127	−0.192
Verbal creativity	0.057	−0.032	0.167	−0.031
Functional outcome	0.092	0.023	0.137	0.112

*p ≤ 0.05; **p ≤ 0.001.

Additionally, correlations between clinical characteristics, neurocognition, social cognition, creativity, and functional outcome were performed (Table 3). As can be seen, functional outcomes correlated positively and significantly with neurocognition (*r* = 0.244, *p* = 0.017) and figural creativity (*r* = 0.270 *p* = 0.008).

**Table 3:** Correlations between clinical characteristics, neurocognition, social cognition, creativity, and functional outcome.

	1	2	3	4	5	6	7	8	9	10	11	12
Duration of illness	1											
Negative symptoms	0.087	1										
Positive symptoms	−0.042	0.045	1									
Disorganization	0.101	0.223*	0.446**	1								
Excited	−0.068	0.052	0.482**	0.321**	1							
Depressed	−0.072	0.207*	0.304*	0.186	0.226*	1						
Neurocognition	−0.291*	−0.175	−0.120	−0.244*	−0.038	−0.105	1					
Social cognition	−0.197	−0.109	−0.001	−0.291*	0.028	0.024	0.527**	1				
Figural creativity	−0.001	−0.284*	−0.107	−0.125	−0.081	0.049	0.233*	0.304*	1			
Figural strengths	−0.021	−0.160	0.021	−0.054	0.051	−0.145	0.245*	0.462**	0.581**	1		
Verbal creativity	−0.064	−0.237*	−0.052	−0.067	−0.166	0.137	0.161	0.298*	0.288*	0.202	1	
Functional outcome	−0.059	−0.173	−0.017	−0.044	−0.058	−0.034	0.244*	0.063	0.270*	0.029	0.052	1

*p ≤ 0.05; **p ≤ 0.001.

### Mediational model explaining functional outcome

A model with path analysis was estimated based on previous literature^1,22,24,40^ and including only paths between the predictors and the mediating or outcome variables that had correlated significantly in the previous analyses. Therefore, positive symptoms, excitement, depression, figural strengths, and verbal creativity were not included in the model. Since sex, age, years of education, and duration of illness correlated significantly with several predictors, these were entered in the model. The fit of the model was very good, SB χ2 (28, *N* = 96) = 32.162, Comparative Fit Index (CFI) = 0.974, Non-Normed Fit Index (NNFI) = 0.959, and Standard Residual Mean Square Root (SRMR) = 0.061.

**Fig. 1:**
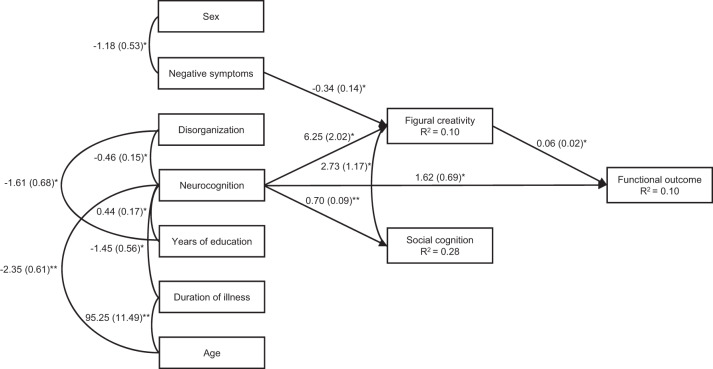
Model of mediation explaining functional outcome through negative symptoms, neurocognition, social cognition, and figural creativity. Given values are non-standardized coefficients with standard errors in parentheses. *p ≤ 0.05; **p ≤ 0.001.

As some of the associations were not statistically significant (the paths from age, years of education and disorganization to social cognition, and the covariance between disorganization and negative symptoms), a new model was estimated only with significant paths. The final model obtained is presented in Fig. 1. As can be seen, on the one hand, neurocognition was positively associated with figural creativity and social cognition, and figural creativity was positively associated with functional outcome. Additionally, social cognition and figural creativity were positively related to each other. On the other hand, negative symptoms were negatively related to figural creativity. Finally, neurocognition was also directly and positively related to functional outcomes. The fit of the model was also very good, SB *χ2* (32, *N* = 96) = 40.445, CFI = 0.954, NNFI = 0.936, and SRMR = 0.073. Next, the significance of the mediational paths was examined via 5,000 bootstrapping samples. The results indicated that figural creativity acted as mediating variable between neurocognition and functional outcome (0.340; 95% confidence interval [0.332, 0.350]) and that figural creativity mediated between negative symptoms and functional outcome (−0.020; 95% confidence interval [−0.021, −0.020]).

In addition, an alternative model was tested in which figural creativity mediated between neurocognition, social cognition, and functional outcome. The Satorra-Bentler’s scaled chi-square difference test^48^ showed that there were not statistically significant differences between the two models [ΔSB χ2 difference (1, *N* = 96) = 0.233, *p* = 0.629]. Although there were not statistically significant differences and the fit indexes where very similar, only the first model was included in the current study.

## Discussion

The aim of this study was to analyze the predictive value of creativity on functional outcome through a mediational model including sociodemographic, clinical, neurocognitive, social cognitive, creativity, and functional outcome variables. To our knowledge, this is the first study analyzing the mediatory role of creativity on functional outcome of patients with schizophrenia. As hypothesized, creativity was significantly associated with functional outcome. More specifically, figural creativity acted as mediator between neurocognition and functional outcome as well as between negative symptoms and functional outcome. Additionally, neurocognition was directly associated with functional outcome. Moreover, although social cognition was not directly related to functional outcome, it was associated with figural creativity and neurocognition. However, contrary to what was expected based on previous literature^1,6,10^, negative symptoms were not directly related to functional outcome and cognitive functioning.

The association found between creativity and functional outcome supports the idea that creative problem solving skills are also applied to real-life problems^27,30^ and therefore, are crucial for daily life functioning. Hence, our findings suggest that patients with schizophrenia who have better creative skills may benefit from this resource to better adapt and cope with their multiple adversities in daily life functioning. As indicated by several authors^31,32^, creativity can be an adaptive ability capable of improving individuals’ both physical and psychological health. In line with our results, few studies have suggested that creativity could influence the functional outcome of individuals with schizophrenia^25^, bipolar disorder^31^, and elderly people^32^.

As expected, creativity was related to neurocognition and social cognition, and mediated the relationship between neurocognition and functional outcome. The association between neurocognition and social cognition with creativity is consistent with previous research carried out in this pathology^38–41^. Similar to this study, another capacity that is related to creativity^43,44^, metacognition capacity, has also been shown to mediate the relationship between neurocognition and functional outcome in schizophrenia^47^.

With respect to the association between cognition and functional outcome, in line with previous studies, neurocognition was strongly associated with functional outcome^6,9,17–19^. However, contrary to other studies^1,23,24^, this association was not mediated by social cognition. In fact, neurocognition was found to be both directly related to functional outcome and through figural creativity. The reason for this may have been that in this study, unexpectedly, social cognition was not directly associated with functional outcome, which could have made the association between neurocognition and functional outcome stronger. A large number of studies have shown a strong relationship between social cognition and functional outcome^1,21–24^. Nevertheless, as in this study, other studies have also found an association between neurocognition and functional outcome, but not with social cognition^9,19^. This may be due to the particular test employed for the measurement of functional outcome. Being a reduced version of the original scale, functional outcome may not have been measured as comprehensively as in other studies^1,23^. Despite the lack of significant results, social cognition should not be underestimated as it has been shown to be a relevant predictor of functional outcome^1,21–24^.

Regarding clinical symptoms, negative symptoms were negatively related to creativity, showing an indirect association with functional outcome through the mediating role of creativity. Other studies carried out on schizophrenia have also found an association between negative symptoms and creativity^39,41^. This makes sense, as some negative symptoms such as avolition can make a creativity task particularly difficult. However, no clinical symptoms were directly related to functional outcome, and only disorganization was related to cognitive functioning. This was an unexpected result, as in general, studies have found a positive association between clinical symptoms and functional outcome in schizophrenia; some of them only with negative symptoms^6,12^, but others with both positive and negative symptoms^1,10,11^ or with disorganization^1^. In contrast, very few studies have found little or even no association between symptoms and functional outcome^49,50^. Additionally, studies have found associations between clinical symptoms and cognitive functioning^1,6,12,13^. In line with this study, Galderisi et al.^1^ found an association between disorganization and neurocognition, which makes sense since this clinical variable is related to attentional capacity and abstract thinking. The lack of significant results overall with clinical symptoms in this study may be partly due to the different scales or the different scoring methods used for the assessment of clinical symptoms and functional outcome across studies. In fact, some of the studies that have used the same scales as in this study have employed a different scoring method^1,11,13,51^. For instance, Bechi et al. used the Positive and Negative Syndrome Scale (PANSS) to assess clinical symptoms, but employed a different scoring method^51^. Galderisi et al.^1^ did use the same scoring method for the positive symptoms and disorganization, but not for the negative symptoms and they used different measures for functional outcome.

Taken together, findings from this study support the well-known idea that cognitive functioning contributes to functional outcome in schizophrenia and point out the specific role of creativity in this association. Additionally, these results also support the association between negative symptoms and functional outcome through creativity. As Richards stated, in the face of adversity, humans have the potential for resilient creative responses^31^. Thus, creativity seems to be a key capacity for the performance of daily life activities and for dealing with real-life challenging situations. These results mark an important milestone and open a new window to future lines of research that could explore other possible factors as determinants of functional outcome in schizophrenia. Indeed, research should not only focus on the negative aspects of the disease, such as clinical symptoms or cognitive impairment, as possible predictors of functional outcome, but there should be a shift towards positive psychology. In fact, in addition to creative capacity, other resources such as resilience or positive humor should be taken into account when studying functional outcome. Regarding resilience, Galderisi et al. already found that resilience has a relevant implication in real-life functioning among patients with schizophrenia^1^. This capacity has been closely related to creativity and has even been suggested that creativity is a potential tool to promote resilience^52^. With respect to humor, this has also been associated with creativity and it is supposed to enhance creativity^53^. Cai et al. found that a humor intervention had positive effects on different domains such as negative symptoms, depression, and anxiety among patients with schizophrenia, although they did not measure functional outcome^54^. Furthermore, the involvement of these kinds of resources on functional outcome should not be limited only to the field of psychosis, but to the whole field of psychopathology as well as to the healthy population.

The importance of this study lies in the fact that understanding which factors underlie functional outcome is vital for the development of more efficient treatment programs. Moreover, the identification of mediators of functional outcome is essential, since these, being closer to the outcome of interest, should become the target of interventions^16^. Specifically, these results provide further evidence for the idea that including training on creative problem-solving skills could be beneficial for the improvement of functional outcome in this disease^37^. Not only in psychosis, but it is suggested that the training or practice of positive resources such as creativity have a positive impact on personal well-being and quality of life of the general population^55,56^. Nevertheless, there are multiple ways of improving creativity. For instance, it could be promoted through “creative-training programs” that include problem-solving or divergent thinking exercises^37,55^. Alternatively, it could be enhanced through creative activities aimed at improving well-being, such as art therapy^55^. Furthermore, creativity could also be trained indirectly through other kind of interventions such as humor intervention or laughter therapy^53^, through cognitive remediation^57^, or by means of transcranial direct current and random noise stimulation^58–60^. In addition, the inclusion of training of positive resources such as creativity or humor in rehabilitation programs could encourage adherence to treatment and motivation, since it may be more interesting for patients. As noted by Cai et al., including training in positive resources could facilitate coping abilities as well as social functioning^54^. Altogether, evidence suggests that integrated and holistic treatments addressing multiple domains such as neurocognition, social cognition, and creativity as well as negative symptoms should be applied in order to improve real-life functioning in mental disorders. In fact, the use of combined and multidisciplinary treatments could produce better results than applying them individually, as they could be mutually reinforcing.

Several limitations should be considered in this study. First, functional outcome was addressed through a social functioning scale which included the assessment of different dimensions of real-world functioning (e.g., interpersonal relations, independent living, and employment); however, the additional measurement of other domains such as quality of life or a more exhaustive assessment of some domains like vocational functioning would have provided a broader picture of the impact of creativity on functional outcome. Second, since this study aimed to explore the role of creativity on daily functioning, creativity assessment could have included the measure of everyday creativity in addition to the creativity tasks employed. Future studies should include this kind of scales to acquire a greater understanding about its role in functional outcomes. Third, though this study included a multidimensional neurocognitive evaluation, it would be interesting for future research to analyze the role of other additional cognitive domains also impaired in schizophrenia such as visual and auditory perception or visual memory, in order to provide a further understanding of the role of neurocognition in creativity in this disease. Fourth, the sample was unbalanced towards men, which may have affected the interpretation of the results. Nevertheless, sex was entered in the mediational model to control for its possible effect. Fifth, the small sample size limited the statistical power of the mediational analysis and prevented the inclusion of individual cognitive dimensions, thus more studies should be conducted with larger samples and findings from this study need to be interpreted with care. Finally, the cross-sectional design of this study did not allow to test causal relationships, so results from the present study should be considered with caution. Future studies could analyze these possible long-term relationships. Bearing these limitations in mind, we believe that this study provides initial and relevant evidence of the mediational role of creativity in daily life functioning in schizophrenia, which has significant implications on the development of interventions aimed at improving functional outcomes. Furthermore, this study opens up a new field of research on additional personal resources as possible determinants of functional outcome in schizophrenia and other diseases.

## Methods

### Participants

Ninety-six patients diagnosed with schizophrenia (85 males and 11 females) were recruited from the Psychiatric Hospital of Álava and the Mental Health Network in Álava (Spain). All patients met the diagnostic criteria for schizophrenia according to the Structured Clinical Interview for DSM-V (the American Psychiatric Association’s Diagnostic and Statistical Manual of Mental Disorders, Fifth Edition)^61^. The mean age of the sample was 41.37 (SD = 10.79) years old and mean years of education was 10.25 (SD = 2.56). 77.08% of the sample were right-handed, 4.16% left-handed, and 18.75% mixed-handed. The 58.3% of the sample were outpatients and 41.7% were inpatients. Mean age of onset of the disease was 22.79 (SD = 5.67), with a mean of previous hospitalizations of 5.84 (SD = 6.31), mean duration of illness of 18.59 (SD = 10.31), and a mean of medication dosage (chlorpromazine equivalent doses - mg/day) of 511.37 (SD = 295.51). The defined daily dose method was used to change medication to chlorpromazine^62,63^.

Exclusion criteria consisted of: (a) clinical instability (meeting the relapse criteria of Csernansky et al.^64^); (b) significant changes in the antipsychotic treatment in the previous three months; (c) cognitive impairment secondary to another disease; (d) diagnosis of an active Major Affective Disorder; and (e) diagnosis of Substance Use Disorder (DSM-V)^61^ including alcohol during the six months prior to study inclusion (with the exception of nicotine). All participants took part voluntarily, providing written informed consent to participate, and they did not receive any monetary reward for taking part in the study. The study protocol was approved by the Clinical Research Ethics Committees of the Autonomous Region of the Basque Country (CEIC-E) in Spain (PI2017044). This study forms part of a larger ongoing project, which is registered in clinicaltrials.gov (NCT03509597).

### Measures

#### Neurocognition

Neurocognition was measured by means of the following tests assessing cognitive flexibility, processing speed, working memory, verbal memory, and inhibition: the number of categories completed and the number of perseverative errors from the Modified Wisconsin Card Sorting Test (M-WCST)^65^; Word, Color, and Word-Color values from the Stroop Color and Word Test^66^; the Backward Digit Span subtest from the Wechsler Adult Intelligence Scale-III (WAIS-III)^67^; the three learning trials and the delayed recall trial from the Hopkins Verbal Learning Test (HVLT version 2)^68^; and the Symbol-Coding subtest from the Wechsler Adult Intelligence Scale-III^67^. All these neurocognitive scores were converted into Z-scores based on the sample of the study. Some scores were adjusted so that higher scores indicated better cognitive performance. A neurocognition composite was then obtained using these Z-scores (Cronbach’s alpha = 0.77).

#### Social cognition

The evaluation of social cognition included the measurement of three domains: theory of mind, social perception, and emotion processing. Theory of mind was assessed by means of the Happé Test “Strange Stories Task”^69^. Four stories from this test were used. Social perception was measured through the Social Attribution Task-Multiple Choice II (SAT-MC-II)^70^. Finally, for the assessment of emotion processing, the Spanish adaptation of the Bell Lysaker Emotion Recognition Test (BLERT)^71^ was used. A composite score of social cognition was obtained from the Z scores of these three tests based on the sample of the study (Cronbach’s alpha = 0.70).

#### Creativity

Figural and verbal creativity were assessed by means of two subtests from the Torrance Test of Creative Thinking^72,73^. Figural creativity was measured through the *Picture Completion* subtest. This task consists of completing ten unfinished pictures by producing as many ideas as possible. The following dimensions were obtained: originality, fluency, flexibility, elaboration, resistance to premature closure, and abstractness of titles. Flexibility was scored using the criteria from the Spanish adaptation of the Torrance Test of Creative Thinking^74^. Total figural creativity score was calculated through the sum of these six dimensions. In addition, a figural creative strengths score was calculated based on the manual^73^. Figural creative strengths consisted of 11 criterion-referenced measures: emotional expressiveness, storytelling articulateness, movement or action, expressiveness of titles, synthesis of incomplete figures, unusual visualization, internal visualization, humor, richness of imagery, colorfulness of imagery, and fantasy.

The *Unusual Uses* subtest was administered in order to assess verbal creativity. In this activity, participants are asked to write as many unusual uses as possible for cardboard boxes. Originality, fluency, and flexibility dimensions were measured. The sum of these three dimensions was used to obtain the total verbal creativity score. Four minutes were given to complete each subtest.

#### Clinical symptoms

The PANSS^75^ was used for the assessment of positive symptoms and general psychopathology. Scores for the dimensions “positive symptoms”, “disorganization”, “excitement”, and “depression” were obtained following the consensus 5-factor solution proposed by Wallwork et al.^76^, which included four, three, four, and three items respectively. As recommended by the NIMH-MATRICS Consensus Statement on Negative Symptoms^77,78^, negative symptoms were measured through the Brief Negative Symptom Scale (BNSS)^79^, composed of 13 items.

#### Functional outcome

Functional outcome was measured using the short Spanish version of the Social Functioning Scale (SFS)^80^. It consists of a 15-item self-report scale that evaluates different functioning domains including social engagement and withdrawal, interpersonal communication, pro-social activities, independence (competence and performance), recreation, and employment.

#### Handedness

Handedness was measured through the Edinburgh Handedness Inventory^81^. The formula (right - left/right + left) was used to calculate handedness consistency and the scores obtained ranged from 100 (wholly right-handed) to −100 (wholly left-handed). Participants who obtained scores ranging from −79 to 79 were considered to be mixed-handed, and those with scores ranging from −100 to −80 or from 80 to 100 to be consistent-handed.

An expert neuropsychologist administered and corrected all the cognitive and creativity tests and clinical symptoms were assessed by expert trained psychiatrists.

### Data analyses

Statistical analyses were carried out by IBM SPSS version 26.0 (SPSS Inc., Chicago, USA). Data were tested for normality using the Kolmogorov-Smirnov test. Missing values were imputed using the expectation maximization (EM) algorithm. Spearman´s Rho, Pearson’s *r* and point-biserial correlations were performed between sociodemographic, clinical, neurocognitive, social cognitive, creative, and functional outcome variables. Significance level was set at 0.05.

To test the mediation hypothesis, path analysis was used with LISREL 9.2^82^. The robust maximum likelihood (RML) method was employed, which requires an estimate of the asymptotic covariance matrix of the variances and covariates of the sample and includes the scaled χ2 Satorra-Bentler index (SB χ2). The goodness of fit of the model was evaluated by CFI, NNFI, and SRMR. According to Hu and Bentler^83^, CFI and NNFI values higher than .90, and SRMR values smaller than .08 reflect a good fit^83^. The Satorra-Bentler’s scaled chi-square difference test^48^ was performed to compare two alternative models, using the specific program designed by Crawford and Henry^84^.

### Reporting Summary

Further information on research design is available in the Nature Research Reporting Summary linked to this article.

## Supplementary information

reporting summary

Supplementary Table 1

## Data Availability

The data that support the findings of this study are available from the corresponding author on reasonable request. The data are not publicly available due to them containing information that could compromise research participant privacy or consent.

## References

[1] Galderisi S (2014). The influence of illness-related variables, personal resources and context-related factors on real-life functioning of people with schizophrenia. World Psychiatry.

[2] Harvey PD (2014). Disability in schizophrenia: contributing factors and validated assessments. J. Clin. Psychiatry.

[3] Fleischhacker WW (2014). Schizophrenia-Time to commit to policy change. Schizophr. Bull..

[4] Harvey PD, Strassnig M (2012). Predicting the severity of everyday functional disability in people with schizophrenia: Cognitive deficits, functional capacity, symptoms, and health status. World Psychiatry.

[5] Bowie CR (2008). Predicting schizophrenia patients’ real-world behavior with specific neuropsychological and functional capacity measures. Biol. Psychiatry.

[6] Ojeda N (2019). An outcome prediction model for schizophrenia: a structural equation modelling approach. Rev. Psiquiatr. Salud Ment..

[7] Green MF (2016). Impact of cognitive and social cognitive impairment on functional outcomes in patients with schizophrenia. J. Clin. Psychiatry.

[8] Lepage M, Bodnar M, Bowie CR (2014). Neurocognition: Clinical and functional outcomes in schizophrenia. Can. J. Psychiatry.

[9] Fu S, Czajkowski N, Rund BR, Torgalsbøen AK (2017). The relationship between level of cognitive impairments and functional outcome trajectories in first-episode schizophrenia. Schizophr. Res..

[10] Fervaha G, Foussias G, Agid O, Remington G (2014). Impact of primary negative symptoms on functional outcomes in schizophrenia. Eur. Psychiatry.

[11] Leifker FR, Bowie CR, Harvey PD (2009). Determinants of everyday outcomes in schizophrenia: The influences of cognitive impairment, functional capacity, and symptoms. Schizophr. Res..

[12] Ventura J, Hellemann GS, Thames AD, Koellner V, Nuechterlein KH (2009). Symptoms as mediators of the relationship between neurocognition and functional outcome in schizophrenia: A meta-analysis. Schizophr. Res..

[13] Lin CH (2013). Clinical symptoms, mainly negative symptoms, mediate the influence of neurocognition and social cognition on functional outcome of schizophrenia. Schizophr. Res..

[14] Milev P, Ho B, Arndt S, Andreasen NC (2005). Predictive values of neurocognition and negative symptoms on functional outcome in schizophrenia: a longitudinal first-episode study with 7-year follow-up. Am. J. Psychiatry.

[15] Fervaha G, Foussias G, Agid O, Remington G (2015). Motivational deficits in early schizophrenia: prevalent, persistent, and key determinants of functional outcome. Schizophr. Res..

[16] Green MF, Horan WP, Lee J (2019). Nonsocial and social cognition in schizophrenia: current evidence and future directions. World Psychiatry.

[17] Green MF, Kern RS, Braff DL, Mintz J (2000). Neurocognitive deficits and functional outcome in schizophrenia: are we measuring the ‘right stuff’?. Schizophr. Bull..

[18] Strassnig MT (2015). Determinants of different aspects of everyday outcome in schizophrenia: the roles of negative symptoms, cognition, and functional capacity. Schizophr. Res..

[19] Peña J (2018). Mechanisms of functional improvement through cognitive rehabilitation in schizophrenia. J. Psychiatr. Res..

[20] Green MF (1996). What are the functional consequences of neurocognitive deficits in schizophrenia?. Am. J. Psychiatry.

[21] Fett AKJ (2011). The relationship between neurocognition and social cognition with functional outcomes in schizophrenia: A meta-analysis. Neurosci. Biobehav. Rev..

[22] Green MF, Llerena K, Kern RS (2015). The ‘right Stuff’ revisited: what have we learned about the determinants of daily functioning in schizophrenia?. Schizophr. Bull..

[23] Sergi MJ, Rassovsky Y, Nuechterlein KH, Green MF (2006). Social perception as a mediator of the influence of early visual processing on functional status in schizophrenia. Am. J. Psychiatry.

[24] Schmidt, S. J., Mueller, D. R. & Roder, V. Social cognition as a mediator variable between neurocognition and functional outcome in schizophrenia: Empirical review and new results by structural equation modeling. *Schizophr. Bull*. **37**, 542–546 (2011). 10.1093/schbul/sbr079 PMC3160114 21860046

[25] Nemoto T, Kashima H, Mizuno M (2007). Contribution of divergent thinking to community functioning in schizophrenia. Prog. Neuro-Psychopharmacol. Biol. Psychiatry.

[26] Sternberg, R. J. & Lubart, T. I. *Defying the crowd: Cultivating creativity in a culture of conformity. Defying the crowd: Cultivating creativity in a culture of conformity*. (Free Press, 1995).

[27] Plucker, J. A., Esping, A., Kaufman, J. C. & Avitia, M. J. Creativity and Intelligence. In *Handbook of intelligence: Evolutionary Theory, Historical Perspective, and Current Concepts* (eds. Goldstein, S., Princiotta, D. & Naglieri, J. A.) 283–291 (2015). 10.1007/978-1-4939-1562-0

[28] Flood M, Scharer K (2006). Creativity enhancement: Possibilities for successful aging. Issues Ment. Health Nurs..

[29] Russ SW (1998). Play, creativity, and adaptive functioning: Implications for play interventions. J. Clin. Child Psychol..

[30] Richards R (1993). Everyday Creativity, eminent creativity, and psychopathology. Psychol. Inq..

[31] Richards, R. Everyday creativity: Process and way of life—Four key issues. in *The Cambridge handbook of creativity*. (eds. Kaufman, J. C. & Sternberg, R. J.) 189–215 (Cambridge University Press, 2010). 10.1017/CBO9780511763205.013

[32] Grebennikova, V., Nikitina, N. & Gardanova, Z. Promoting leadership and creativity among elderly people. in *4th International Conference on Social, Business, and Academic Leadership* 359, 77–81 (Atlantis Press, 2019).

[33] Rindermann H, Neubauer AC (2004). Processing speed, intelligence, creativity, and school performance: Testing of causal hypotheses using structural equation models. Intelligence.

[34] Acar S, Chen X, Cayirdag N (2017). Schizophrenia and creativity: A meta-analytic review. Schizophr. Res..

[35] Nemoto T, Mizuno M, Kashima H (2005). Qualitative evaluation of divergent thinking in patients with schizophrenia. Behav. Neurol..

[36] Jolly, J. Nonverbal creative abilities in cognition as predictors of coping response patterns in schizophrenia and in schizoaffective and bipolar disorders. (University of San Francisco, 2000).

[37] Nemoto T (2009). Cognitive training for divergent thinking in schizophrenia: A pilot study. Prog. Neuro-Psychopharmacol. Biol. Psychiatry.

[38] Abraham A, Windmann S, McKenna P, Güntürkün O (2007). Creative thinking in schizophrenia: the role of executive dysfunction and symptom severity. Cogn. Neuropsychiatry.

[39] Jaracz J, Patrzała A, Rybakowski JK (2012). Creative thinking deficits in patients with schizophrenia: Neurocognitive correlates. J. Nerv. Ment. Dis..

[40] Sampedro A (2019). Mediating role of cognition and social cognition on creativity among patients with schizophrenia and healthy controls: revisiting the shared vulnerability model. Psychiatry Clin. Neurosci..

[41] Sampedro A (2020). Neurocognitive, social cognitive, and clinical predictors of creativity in schizophrenia. J. Psychiatr. Res..

[42] Carson SH (2011). Creativity and psychopathology: a shared vulnerability model. [Review]. J. Psychiatry - Rev. Can. Psychiatr..

[43] Lizarraga MLSdeA, Baquedano MTSdeA (2013). How creative potential is related to metacognition. Eur. J. Educ. Psychol..

[44] Erbas AK, Bas S (2015). The contribution of personality traits, motivation, academic risk-taking and metacognition to the creative ability in mathematics. Creat. Res. J..

[45] Davies G, Greenwood K (2018). A meta-analytic review of the relationship between neurocognition, metacognition and functional outcome in schizophrenia. J. Ment. Heal..

[46] Lysaker PH (2011). Metacognition and social function in schizophrenia: Associations of mastery with functional skills competence. Schizophr. Res..

[47] Davies G, Fowler D, Greenwood K (2017). Metacognition as a mediating variable between neurocognition and functional outcome in first episode psychosis. Schizophr. Bull..

[48] Satorra A, Bentler P (2001). A scaled difference chi-square test statistic for moment structure analysis. Psychometrika.

[49] Velligan DI (1997). The functional significance of symptomatology and cognitive function in schizophrenia. Schizophr. Res..

[50] Harvey PD (1998). Symptoms, cognitive functioning, and adaptive skills in geriatric patients with lifelong schizophrenia: A comparison across treatment sites. Am. J. Psychiatry.

[51] Bechi M (2017). Exploring functioning in schizophrenia: Predictors of functional capacity and real-world behaviour. Psychiatry Res..

[52] Metzl ES, Morrell MA (2008). The role of creativity in models of resilience: Theoretical exploration and practical applications. J. Creat. Ment. Heal..

[53] Chen, C.-H. H., Chen, H.-C. C. & Roberts, A. M. Why Humor Enhances Creativity From Theoretical Explanations to an Empirical Humor Training Program: Effective “Ha-Ha” Helps People to “A-Ha”. in *Creativity and Humor* (eds. Luria, S. R., Baer, J. & Kaufman, J. C.) 83–108 (Academic Press, 2019). 10.1016/B978-0-12-813802-1.00004-1

[54] Cai C, Yu L, Rong L, Zhong H (2014). Effectiveness of humor intervention for patients with schizophrenia: a randomized controlled trial. J. Psychiatr. Res..

[55] Forgeard, M. J. C. & Eichner, K. V. Creativity as a target and tool for positive interventions. in *Handbook of Positive Psychological Interventions* (eds. Parks, A. C. & Schueller, S. M.) 137–154 (Wiley-Blackwell, 2014).

[56] Conner TS, DeYoung CG, Silvia PJ (2018). Everyday creative activity as a path to flourishing. J. Posit. Psychol..

[57] Kiritsis, P. Preserving the creative advantages of schizophrenia: a quantitative pretest-posttest study on the effects of cognitive remediation training on creativity. (Sofia University, 2018).

[58] Peña J, Sampedro A, Bilbao NI, Elorza LZ (2020). The effect of transcranial random noise stimulation (tRNS) over bilateral posterior parietal cortex on divergent and convergent thinking. Sci. Rep..

[59] Peña J, Sampedro A, Ibarretxe-Bilbao N, Zubiaurre-Elorza L, Ojeda N (2019). Improvement in creativity after transcranial random noise stimulation (tRNS) over the left dorsolateral prefrontal cortex. Sci. Rep..

[60] Lucchiari C, Sala PM, Vanutelli ME (2018). Promoting creativity through transcranial direct current stimulation (tDCS). A critical review. Front. Behav. Neurosci..

[61] American Psychiatric Association. *Diagnostic and Statistical Manual of Mental Disorders (5th ed.)*. (American Psychiatric Association, 2013).

[62] Leucht S, Samara M, Heres S, Davis JM (2016). Dose equivalents for antipsychotic drugs: the DDD method. Schizophr. Bull..

[63] Rothe PH, Heres S, Leucht S (2018). Dose equivalents for second generation long-acting injectable antipsychotics: The minimum effective dose method. Schizophr. Res..

[64] Csernansky JG, Mahmoud R, Brenner R (2002). A comparison of risperidone and haloperidol for the prevention of relapse in patients with schizophrenia. N. Engl. J. Med..

[65] Schretlen, D. *Modified Wisconsin Card Sorting Test professional manual*. (2010).

[66] Golden, C. J. *STROOP: Test de colores y palabras*. (2010).

[67] Wechsler, D. *WAIS-III Manual: Wechsler Adult Intelligence Scale-III*. (1997).

[68] Brandt, J. & Benedict, R. *Hopkins Verbal Learning Test-Revised*. (Psychological Assessment Resources, 2001).

[69] Happé FG (1994). An advanced test of theory of mind: understanding of story characters’ thoughts and feelings by able autistic, mentally handicapped, and normal children and adults. J. Autism Dev. Disord..

[70] Johannesen JK, Lurie JB, Fiszdon JM, Bell MD (2013). The social attribution task-multiple choice (SAT-MC): a psychometric and equivalence study of an alternate form. ISRN Psychiatry.

[71] Bell M, Bryson G, Lysaker P (1997). Positive and negative affect recognition in schizophrenia: a comparison with substance abuse and normal control subjects. Psychiatry Res..

[72] Torrance, E. P. *The Torrance Tests of Creative Thinking — Norms-Technical Manual Research Edition—Verbal Tests, Forms A and B— Figural tests, Forms A and B*. (Personnel Press, 1966).

[73] Torrance, E. P. *Torrance Tests of Creative Thinking*. (Scholastic Testing Service. Inc., 2016).

[74] Jiménez, J. E., Artiles, C., Rodríguez, M. & García, E. *Adaptación y baremación del test de pensamiento creativo de Torrance: expresión figurada*. (2007).

[75] Kay SR, Fiszbein A, Opler LA (1987). The positive and negative syndrome scale (PANSS) for schizophrenia. Schizophr. Bull..

[76] Wallwork RS, Fortgang R, Hashimoto R, Weinberger DR, Dickinson D (2012). Searching for a consensus five-factor model of the Positive and Negative Syndrome Scale for schizophrenia. Schizophr. Res..

[77] Carpenter, W. T., Blanchard, J. J. & Kirkpatrick, B. New Standards for Negative Symptom Assessment. *Schizophrenia Bulletin* (2016). 10.1093/schbul/sbv160 10.1093/schbul/sbv160 PMC4681567 26615186

[78] Kirkpatrick, B., Fenton, W. S., Carpenter, W. T. & Marder, S. R. The NIMH-MATRICS consensus statement on negative symptoms. in *Schizophrenia Bulletin* (2006). 10.1093/schbul/sbj053 10.1093/schbul/sbj053 PMC2632223 16481659

[79] Kirkpatrick B (2011). The brief negative symptom scale: psychometric properties. Schizophr. Bull..

[80] Alonso J (2008). Desarrollo y validación de la versión corta de la Escala de Funcionamiento Social en esquizofrenia para su uso en la práctica clínica. Actas Esp. Psiquiatr..

[81] Oldfield RC (1971). The assessment of handedness: the Edinburgh inventory. Neuropsychologia.

[82] Jöreskog, K. & Sörbom, D. LISREL 9.20 for Windows [Computer software]. (2015).

[83] Hu LT, Bentler PM (1999). Cutoff criteria for fit indexes in covariance structure analysis: conventional criteria versus new alternatives. Struct. Equ. Model..

[84] Crawford JR, Henry JD (2003). The depression anxiety stress scales (DASS): normative data and latent structure in a large non-clinical sample. Br. J. Clin. Psychol..

