# Supplementary material for: The impact of creativity on functional outcome in schizophrenia: a mediational model
Source: NPJ Schizophr. 2021 Feb 26;7:14. doi: 10.1038/s41537-021-00144-5 (PMC7910291; doi:10.1038/s41537-021-00144-5)
Supplement: Supplementary file 2 — Supplementary Table 1 [file 41537_2021_144_MOESM2_ESM.pdf]

Supplementary Table 1. Correlations between neurocognitive domains, social cognitive domains, creativity and functional outcome.

	1	2	3	4	5	6	7	8	9	10	11	12
1. Cognitive flexibility	1											
2. Processing speed	.073	1										
3. Working memory	.178	.328**	1									
4. Verbal memory	.216*	.318*	.179	1								
5. Inhibition	.060	.447**	.234*	.209*	1							
6. Theory of mind	.263*	.338**	.176	.334**	.241*	1						
7. Social perception	.309*	.189	.245*	.335**	.207*	.381**	1					
8. Emotion processing	.246*	.348**	.161	.221*	.251*	.486**	.371**	1				
9. Figural creativity	.024	.337**	.169	.026	.139	.190	.305*	.279*	1			
10. Figural strengths	.140	.278*	.130	.107	.105	.344**	.316*	.452**	.581**	1		
11. Verbal creativity	.041	.186	.075	.113	.016	.138	.361**	.185	.288*	.202	1	
12. Functional outcome	.157	.239*	.140	.071	.276*	-.066	.187	.151	.270*	.029	.052	1

* $p \leq .010$; ** $p \leq .001$.
